# Cross-Sectional and Time-Dependent Analyses on Inflammatory Markers following Natural Killer Cell Activity

**DOI:** 10.3390/diagnostics12020448

**Published:** 2022-02-09

**Authors:** Yun-Kyong Lee, Ji-Hee Haam, Sung-Hoon Cho, Young-Sang Kim

**Affiliations:** 1 Chaum Life Center, CHA University, Seoul 06062, Korea; ykleefm@chamc.co.kr (Y.-K.L.); hamjhi@chamc.co.kr (J.-H.H.); 2 TS Bio, Seoul 08389, Korea; nk8275@naver.com; 3 Department of Family Medicine, CHA Bundang Medical Center, CHA University, Seongnam 13496, Korea

**Keywords:** natural killer cell activity, inflammation, neutrophil-to-lymphocyte ratio, immunity

## Abstract

The function of natural killer (NK) cells in inflammation has not been explored enough in large-scale population studies. The cross-sectional and time-dependent relationship between NK cell activity (NKA) and inflammatory markers was examined. Methods: A total of 7031 subjects were involved in the cross-sectional analyses. Non-linear relationship between NKA and inflammatory indices was analyzed using generalized additive models. The time-dependent changes were analyzed in 1005 subjects with repeated measurement in 3–6 months. The changes in inflammatory markers were analyzed based on the changes in NKA. Results: As NKA reduces to a very low level, the white blood cell (WBC) and neutrophil counts increase sharply, and the lymphocyte count exhibits a slow decline. With increasing NKA larger than about 500 pg/mL, WBC and neutrophil-lymphocyte ratio (NLR) reduces in a mild slope. Among the subjects with repeated measurements, the follow-up NKA was increased with advancing baseline NKA levels. The subjects with a reduction in NKA indicated increment in WBC count, neutrophil count, and NLR, and decrease in lymphocyte count. Conclusions: Very low levels of NKA suggest a high inflammatory immune response. The changes in NKA may interact with the balance between neutrophils and lymphocytes.

## 1. Introduction

Natural killer (NK) cells are large granular lymphocytes that play critical roles in innate immunity because they recognize and remove virus-infected and neoplastic cells [1]. NK cells have cytotoxic activity regulated by activation and inhibition of surface receptors and antibodies [2]. Activated NK cells release cytotoxic granules and secrete cytokines, such as interferon (IFN)-γ and tumor necrosis factor (TNF)-α; these cytokines play an immunoregulatory role as they activate NK cells and further promote cytokine secretion [3]. By these means, NK cells impact the development of adaptive immune responses [4] and contribute to progression and resolution of diseases [5]. In contrast, NK cells can also cause excessive inflammation or even autoimmunity [6].

Established methods that measure NKA, such as ^51^Cr release assay and CD107a degranulation assay, have been widely used to determine NK cell function; however, these methods are complicated and time-consuming because they need the isolation of peripheral blood mononuclear (PBMC) or NK cells [7]. To overcome these challenges, a relatively simple assay that uses whole blood instead of PBMC or isolated NK cells was recently developed for commercial use to measure the activity of NK cells. This novel assay uses the serum of ex vivo-stimulated whole blood to detect secreted IFN-γ from NK cells as an indicator of NKA [8]. Using this method, clinical studies have indicated that NKA is a useful marker for different cancers and is related to immunologic conditions such as aging and vitamin D insufficiency [9,10,11,12].

Many peripheral blood indices, including complete blood count (CBC), erythrocyte sedimentation rate (ESR), and level of C-reactive protein (CRP), are widely used as inflammatory markers [13,14]. It has been reported that neutrophils play an opposite role in the function of NK cells; while neutrophils inhibit NK cell functions [15], they are also needed for proper NK cell maturation [16]. The neutrophil-to-lymphocyte ratio (NLR) is an attractive biomarker of systemic inflammation because it is easily calculated from the absolute neutrophil and lymphocyte counts collected from a routine complete blood cell count with differentials [17]. NLR as an inflammatory marker also has prognostic value for cardiovascular diseases, cancers, and COVID-19 [18,19,20]. Although a previous study assessed the relationship between NKA and inflammatory markers, especially NLR [21], the number of subjects was relatively small, and the range of NKA distribution was narrow. As of yet, large-scale analysis of the relationship between NKA and inflammatory indices has not been performed, and the change in inflammatory markers according to the time-dependent change in NKA has not been analyzed. This study assessed the non-linear association between NKA and common inflammatory indices. Additionally, the association between changes in NKA and the subsequent changes in inflammatory indices was also investigated.

## 2. Subjects and Methods

### 2.1. Study Population

This study was conducted based on data from Chaum Life Center. The data were obtained from the medical records of outpatient clinics and health checkups between 2016 and 2020. The records containing coincident conduction of CBC and NKA were included in this study. Nine thousand five hundred and fifty samples were obtained from outpatient clinics (N = 2625) and health checkups (N = 6925). The samples were collected from one or multiple visits of 3098 men and 3933 women. The cross-sectional analyses were conducted using the baseline data from the subjects.

To analyze the time-dependent difference of CBC parameters and inflammatory indices according to NKA changes, the subjects who had undergone two or more tests with intervals between 90 and 180 d were extracted. One thousand one hundred and fifteen subjects met this criterion. Those who had a history of any malignancies or steroid use were excluded (N = 110). Finally, 1005 subjects were included in the analyses for the study on time-dependent changes.

The study was conducted following the guidelines of the Helsinki Declaration and approved by the Institutional Review Board of CHA Bundang Medical Center (CHAMC 2020-10-006).

### 2.2. Medical History of the Subjects

The medical histories and lifestyle habits were obtained from the subjects. Subjects were categorized as nonsmokers or current smokers based on their smoking habits. Significant alcohol consumption was defined as >21 standard drinks/week in men and >14 standard drinks/week in women over 2 years.

### 2.3. Measurements and Blood Sampling

Height and weight were measured in a standing position without shoes and were recorded to the first decimal point in centimeters and kilograms, respectively. Body mass index was defined as body weight in kilograms divided by the height squared in meters.

Blood samples were obtained in the morning after the patient had fasted overnight for at least eight hours and derived from the antecubital area. Serum samples were stored at 4 °C and analyzed within a day of sampling. The neutrophil and lymphocyte counts were estimated as each proportion of the number of WBCs. NLR is defined as neutrophil count divided by lymphocyte count. CRP was measured using an automatic analyzer (Hitachi 7600; Hitachi, Tokyo, Japan). ESR was analyzed with photometric capillary stopped flow kinetic analysis (TEST1; Alifax SpA, Polverara, Italy).

### 2.4. NKA Measurement

NKA was measured using a recently developed blood test (NK Vue^®^ Kit, NKMAX, Sungnam, Korea). A 1 mL sample of whole blood, obtained using a direct vacutainer system from a new venipuncture point, was directly transferred into a specific tube for NKA tests. The blood collection tube for analyzing NKA contains a patented stimulatory cytokine (Promoca^®^, NKMAX, Sungnam, Korea). The collection tube was gently and repeatedly mixed; within 30 min of collection, the tube was incubated for 20−24 h in a 37 °C chamber, following the manufacturer’s instructions. During the incubation period, the stimulatory cytokine causes the secretion of IFN-γ into the plasma; this secretion predominantly occurs through NK cells rather than other innate or adaptive immune cells [8,22]. The supernatant was obtained and centrifuged at 3000× *g* for three minutes following incubation. The supernatant was loaded onto enzyme-linked immunosorbent assay (ELISA) plates. Using a designed ELISA, IFN-γ levels were quantitated in pg/mL.

This study used reference ranges given by the test-kit manufacturer [23] and defined <100 as extremely low (group 1), 100–250 (group 2) as very low, 250–500 (group 3) as low, and ≥500 as normal. Then, those defined as normal were further classified into 500–1000 (group 4), 1000–2000 (group 5), and ≥2000 (group 6) to exhibit the distribution efficiently.

### 2.5. Statistical Analysis

The general features were expressed as means ± SD, median (interquartile range), or number (proportion).

Among the cases from the 1005 subjects with two or more measurements, the values at baseline were compared with those measured at the nearest time from the time of a year after the baseline. The repeated measured values were expressed as median (interquartile range) and compared using the Wilcoxon signed rank test.

Smoothing splines were drawn using generalized additive models to assess the non-linear relationship between NKA and CBC parameters and inflammatory indices. Then, the cases were classified into six groups following the NKA values shown as above. WBC, CRP, and NLR were determined as high when WBC ≥ 10,000, CRP ≥ 3 g/L, and NLR ≥ 4 [24,25]. The proportion of high level in three indices was calculated following the NKA groups, and the calculation was repeated following the sex and age groups (<50 vs. ≥50 years). *p* for trend in the ordered groups was estimated using Cochran–Armitage tests.

Among the subjects with repeated measurements, the NKA value at follow-up and NKA change from baseline was estimated according to baseline NKA groups. To assess the association between NKA change and the change of CBC parameters and inflammatory indices, the mean of the changes in the parameters was calculated following the quintile of NKA change. The difference among the quintiles was calculated using ANOVA tests.

All statistical analyses were conducted using the SPSS statistical package, version 26 (IBM, Armonk, NY, USA). Results with *p* < 0.05 were considered statistically significant. The smoothing splines were drawn using R packages 3.62 (R Foundation for Statistical Computing; Vienna, Austria) and R studio 1.2.5033 (PBC; Boston, MA, USA) with gglot2 library.

## 3. Results

### 3.1. Characteristics of Subjects

The baseline features of the subjects are indicated in Table 1. In the 7031 subjects at baseline, the mean age at baseline is 49.1 ± 12.5 years. The median count of WBC, neutrophils, and lymphocytes were 5390, 2928, and 1831/µL, respectively. The median NLR, CRP, and ESR values were 1.59, 0.7 g/L, and 6 mm/h, respectively.

Among 1005 subjects with repeated measurements, the median of WBC and lymphocyte counts was changed from 5400–5290 (*p* = 0.013) and from 1831–1822/µL (*p* = 0.007), respectively. However, changes in NKA, neutrophil count, NLR, and ESR were insignificant.

### 3.2. Association of NKA with CBC Parameters and Inflammatory Indices

**Figure 1:**
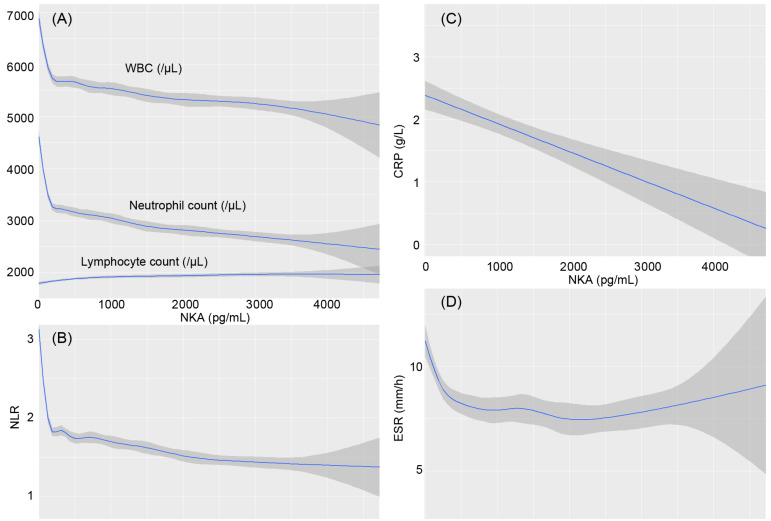
Smoothing splines of the inflammatory indices following natural killer cell activity. NKA, natural killer cell activity; WBC, white blood cell; NLR, neutrophil-to-lymphocyte ratio, CRP, C-reactive protein; ESR, erythrocyte sedimentation rate. The inflammatory indices are (**A**) the parameters in complete blood cell counts, (**B**) neutrophil-to-lymphocyte ratio, (**C**) C-reactive protein, and (**D**) erythrocyte sedimentation rate.

The smoothing splines were drawn using generalized additive models (Figure 1). As NKA reduces to a very low level, less than about 250 pg/mL, the WBC and neutrophil counts increase sharply, and the lymphocyte count exhibits slow decline. Accordingly, as NKA reduces, NLR also increases sharply. Simultaneously, CRP and ESR also increase with a decrease in NKA. In contrast, WBC and NLR decrease in a mild slope with increasing NKA larger than about 500 pg/mL.

**Figure 2:**
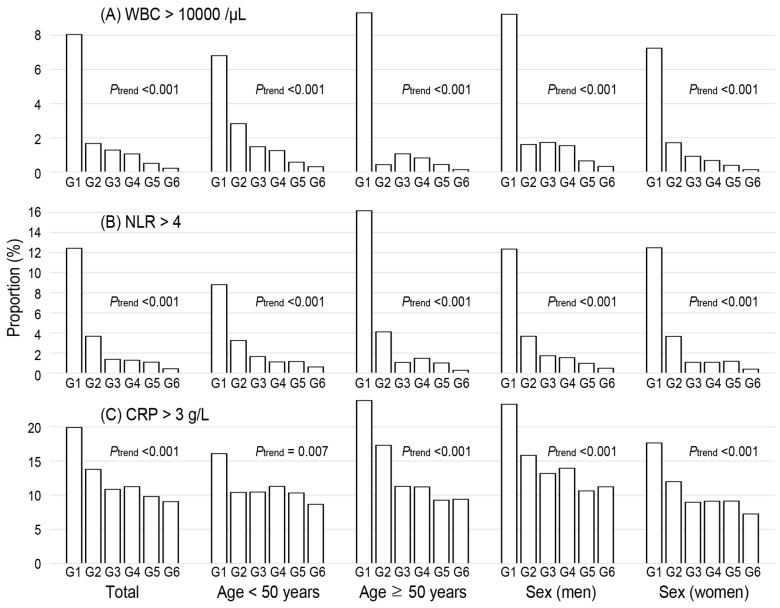
The proportion of high level of inflammatory markers according to the classification of baseline natural killer cell activity. G1–6 represents natural killer cell activity classification (G1, <100; G2, 100–250; G3, 250–500; G4, 500–1000; G5, 1000–2000; G6, ≥2000). The high level of inflammatory markers are defined as (**A**) white blood cell more than 10,000/μL, (**B**) neutrophil-to-lymphocyte ratio more than 4, and (**C**) C-reactive protein more than 3 g/L. *p* for trend was estimated using Cochran–Armitage tests. WBC, white blood cell; NLR, neutrophil-to-lymphocyte ratio, CRP, C-reactive protein.

The probability of high values in WBC count (≥10,000), CRP (≥3 g/L), and NLR (≥4) was calculated in Figure 2. The probability of a high WBC count was 8.0%, 1.7%, and 1.3% in NKA groups 1, 2, and 3, respectively. Similarly, the probability of a high NLR was 12.4%, 3.7%, and 1.4% in groups 1, 2, and 3, respectively. In contrast, the probability of high CRP levels declines slowly with the advance in NKA groups. The patterns are similarly repeated in analyses according to sex and age groups divided by age 50.

### 3.3. The Time-Dependent Changes of NKA and Concomitant Change of CBC Parameters and Inflammatory Indices

**Figure 3:**
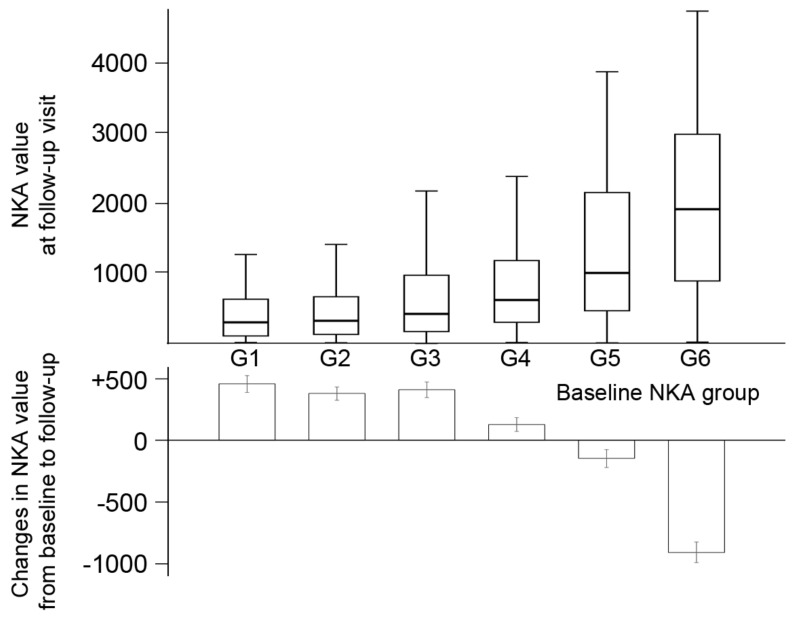
The changes in natural killer cell activity according to the baseline natural killer cell activity classification. Box plots show the median, interquartile range, and minimum/maximum (except for outliers) of NKA at follow-up visit. Bar charts show the mean of the changes in NKA, and the error bars express standard errors. G1–6 represents natural killer cell activity classification (G1, <100; G2, 100–250; G3, 250–500; G4, 500–1000; G5, 1000–2000; G6, ≥2000). NKA, natural killer cell activity.

Among 1005 subjects with repeated measurements, the follow-up NKA was increased with advancing baseline NKA groups (Figure 3). However, in three groups with low NKA, the NKA changes were positive (431.4, 400.0, and 414.6 in groups 1–3, respectively). In contrast, the NKA changes in groups 5 and 6 were −114.8 and −897.8, respectively.

**Figure 4:**
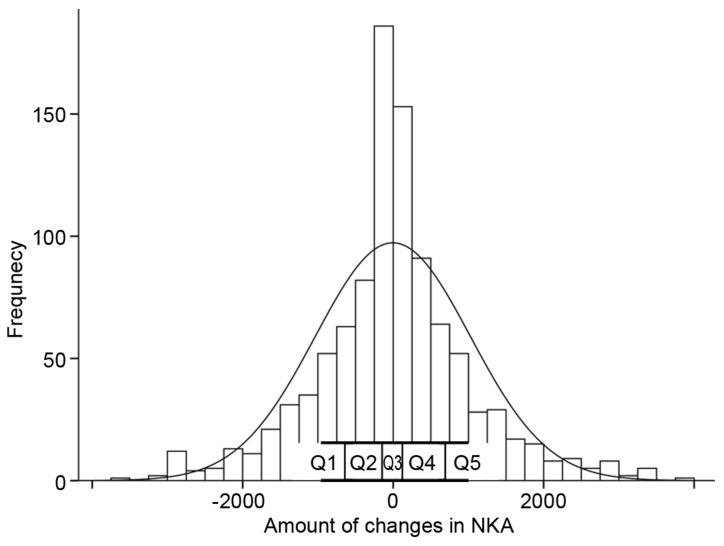
The distribution of the natural killer cell activity changes. Q1–5 represents quintiles of change amount in natural killer cell activity. NKA, natural killer cell activity.

**Figure 5:**
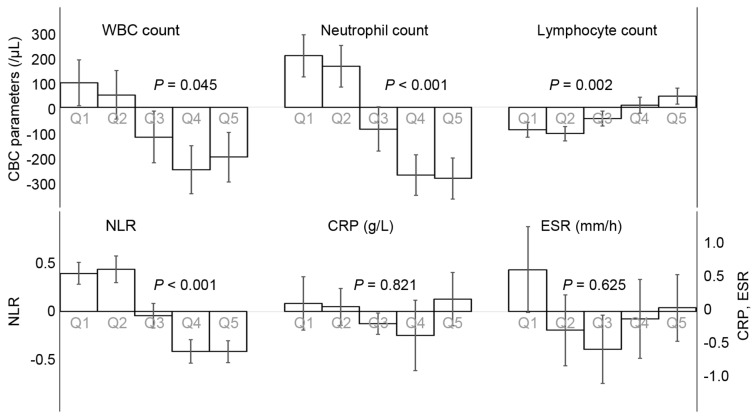
The changes in inflammatory markers according to the quintile of changes in natural killer cell activity. Each bar shows means and error bars express standard errors of means. *p* values were calculated using ANOVA tests. CBC, complete blood count; WBC, white blood cell; NLR, neutrophil-to-lymphocyte ratio, CRP, C-reactive protein; ESR, erythrocyte sedimentation rate.

The NKA changes were classified into quintiles, and the parameter changes were analyzed according to the quintiles (Figure 4). The subjects with a reduction in NKA (Q1 and Q2) exhibited increments in WBC, neutrophil counts, and NLR, and decrement in lymphocyte count (Figure 5). The subjects with an increase in NKA (Q3 and Q4) exhibited the opposite patterns compared to those with the reduction in NKA. According to the change in NKA, the changes in ESR and CRP were varied from one another and insignificant.

## 4. Discussion

This study exhibited a non-linear association between NKA and inflammatory indices. A low level of NKA was strongly related to a high level of inflammatory indices and the probability of inflammation, defined by a high level of WBC, CRP, and NLR. Additionally, within our knowledge, no report was published on the time-dependent changes in NKA and subsequent changes in inflammatory indices. Changes in NKA were associated with changes in CBC parameters, including NLR, but not with changes in CRP and ESR.

A previous study has indicated inverse association between NKA and NLR [21]. Our study showed a similar association, but it was close to a negatively exponential curve. WBC count and NLR were markedly elevated as NKA was being reduced under the very low level. In the previous study, since the subjects with very low levels of NKA were rare, the association between NKA and WBC was not clearly confirmed in them [21]. Our study found that a low level of NKA, especially lower than 100 pg/mL, strongly indicates leukocytosis and high NLR. As is known, CRP and ESR are also critical inflammatory markers. ESR was sharply elevated in those with very low NKA. CRP levels were gradually reduced as NKA was increased. Similarly, the number of CD56bright NK cells negatively correlated with CRP levels [26]. Thus, a higher level of NKA was an index of lower inflammatory state, and a very low NKA level may suggest a strong marker for inflammation.

The proportion of leukocytosis and NLR ≥ 4 was particularly high among those with NKA < 100 pg/mL, suggesting that high NLR and low NKA could be strongly associated with each other in the immune system. IFN-γ secreted by NK cells shapes the Th1 immune response [27] and activates antigen presenting cells and macrophages [28]. Thus, NK cells have cytotoxic immune response to viral- and malignant-transformed cells [29,30]. Decrease in NK cell function may lead to defect protective action on inflammatory changes. Evidence has indicated that NLR and NK cell functions are associated with different inflammation-related disorders, including cancer, COVID-19, and cardiovascular disorders. Firstly, both indices are the prognostic factor of multiple cancers. NLR is recognized as an independent prognostic value in patients with various cancers [15,25,31]. NLR is also associated with overall survival in many solid tumors [25]. Simultaneously, reduced NK cell function in immunologically normal individuals is associated with increased risk of cancer development [9,10,23,32]. Secondly, both NLR and NK cell functions are associated with COVID-19. NLR is a predictive factor for clinical features of COVID-19 [33,34]. Impaired NK cell counts and cytolytic activity were seen to be lowered in patients with severe COVID-19 [35]. The restoration of NK cell effector function has the potential to correct the delicate immune balance in COVID-19 [36]. Thirdly, NLR and NK cell functions are associated with cardiovascular disease (CVD). NLR is a predictor of all-cause mortality and cardiovascular events [20]. NKA was seen to be reduced in patients with coronary artery disease [37]. Clinical abnormalities in the numbers and functions of NK cells are observed in various heart diseases [38].

Our study is the first attempt to show longitudinal NKA changes and its consequent sequelae of inflammatory markers. NKA changes were distributed nearly in the shape of a normal distribution. The amount of change in NKA exhibited a strong linkage with the amount of change in CBC parameters including NLR. The reduction in NKA was clearly associated with the rise in NLR. This close relationship between changes in NKA and NLR follows the common factors in the immune system and inflammation, as mentioned above. However, the changes in ESR and CRP were not significantly correlated with the changes in NKA. Generally, CRP is not a screening test for wellness and should only be used to diagnose and monitor a patient suspected of an inflammatory process [39]. In terms of inflammation, CRP is recognized as a superior marker of WBC [40]. In a study on the mortality of the older population, WBC count and CRP levels independently predict mortality [41]. The association between increasing WBC count and mortality remained significant after adjusting CRP levels. The long-term value of NKA on mortality and morbidity requires further investigation.

Our study has several constraints. Firstly, the study was conducted in a single center; the result of this study may not be generalized worldwide. However, this study included a large sample of the population and a large number of assays, including repeated measurements. The large sample enabled us to show the negatively exponential relationship between NKA and CBC parameters such as NLR. Secondly, NKA was measured using only IFN-γ assays. Although NK cell count was not measured in this study, the methods used are more appropriate for analyses on a large number of samples. Additionally, the effect of Promoca^®^ on NK cells was confirmed [8]. Thirdly, although the medical histories were taken for those with repeated measurements, they could not be collected from all subjects. Malignancies or CVD may affect the measured values, including NKA. This point was considered in the analyses on the repeated measurements. The subjects in our study might mimic the population under various medical conditions.

In conclusion, very low NKA is strongly associated with systemic inflammatory status. The levels and changes of NKA may be influenced by the balance between neutrophils and lymphocytes. NKA is a clinically significant index reflecting immune function and inflammatory status. Further studies should investigate the long-term health issues following the immune status assessed using NKA.

## Figures and Tables

**Table 1:** General characteristics of the subjects.

Subjects at Baseline			
	Total (N = 7031)	Men (N = 3098)	Women (N = 3933)
Age (years)	49.1 ± 12.5	50.7 ± 12.3	47.9 ± 12.5
Body mass index (kg/m^2^)	23.49 ± 3.75	25.3 ± 3.3	21.9 ± 3.4
NKA (pg/mL)	674 (248–1656)	683 (254–1677)	664 (240–1648)
WBC (/µL)	5390 (4500–6490)	5600 (4700–6713)	5230 (4360–6290)
Neutrophil count (/µL)	2928 (2301–3715)	3019 (2409–3802)	2851 (2228–3621)
Lymphocyte count (/µL)	1831 (1499–2218)	1886 (1532–2282)	1789 (1468–2162)
NLR	1.59 (1.24–2.07)	1.59 (1.25–2.07)	1.59 (1.23–2.08)
CRP (g/L)	0.7 (0.4–1.5)	0.9 (0.5–1.8)	0.6 (0.3–1.3)
ESR (mm/h)	6.0 (3.0–11.0)	4.0 (2.0–9.0)	7.0 (3.0–13.0)
Subjects with repeated measurement (N = 1005)			
	Baseline	Follow-up	*p*
Age (years)	51.6 ± 12.0		
Sex (men)	478 (47.6%)		
Hypertension	177 (17.6%)		
Diabetes	58 (5.8%)		
Dyslipidemia	237 (23.6%)		
Alcohol consumer	399 (39.7%)		
Smoking			
Non-smoker	673 (67.0%)		
Current smoker	123 (12.2%)		
Ex-smoker	209 (20.8%)		
NKA (pg/mL)	681 (252–1692)	667 (249–1576)	0.616
WBC (/µL)	5400 (4450–6415)	5290 (4445–6325)	0.013
Neutrophil count (/µL)	2905 (2244–3695)	2870 (2249–3676)	0.065
Lymphocyte count (/µL)	1831 (1519–2230)	1822 (1509–2160)	0.007
NLR	1.59 (1.22–2.04)	1.60 (1.23–2.03)	0.681
CRP (g/L)	0.6 (0.3–1.3)	0.7 (0.4–1.4)	0.037
ESR (mm/h)	6.0 (3.0–11.0)	6.0 (3.0–11.0)	0.917

Data are presented as mean ± SD, median (interquartile range), or number (proportion). *p* values are calculated by Wilcoxon signed rank test. NKA, natural killer cell activity; WBC, white blood cell; NLR, neutrophil-to-lymphocyte ratio, CRP, C-reactive protein; ESR, erythrocyte sedimentation rate.

## Data Availability

Not applicable.

## References

[1] Bryceson Y.T., Chiang S., Darmanin S., Fauriat C., Schlums H., Theorell J., Wood S.M. (2011). Molecular mechanisms of natural killer cell activation. J. Innate Immun..

[2] Smyth M.J., Hayakawa Y., Takeda K., Yagita H. (2002). New aspects of natural-killer-cell surveillance and therapy of cancer. Nat. Cancer.

[3] Bryceson Y., March M., Ljunggren H.-G., Long E.O. (2006). Activation, coactivation, and costimulation of resting human natural killer cells. Immunol. Rev..

[4] Fauriat C., Long E.O., Ljunggren H.-G., Bryceson Y. (2010). Regulation of human NK-cell cytokine and chemokine production by target cell recognition. Blood.

[5] Tosello-Trampont A., Surette F.A., Ewald S.E., Hahn Y.S. (2017). Immunoregulatory Role of NK Cells in Tissue Inflammation and Regeneration. Front. Immunol..

[6] Zitti B., Bryceson Y.T. (2018). Natural killer cells in inflammation and autoimmunity. Cytokine Growth Factor Rev..

[7] Valiathan R., Lewis J.E., Melillo A.B., Leonard S., Ali K.H., Asthana D. (2012). Evaluation of a flow cytometry-based assay for natural killer cell activity in clinical settings. Scand. J. Immunol..

[8] Lee S.-B., Cha J., Kim I.-K., Yoon J.C., Lee H.J., Park S.W., Cho S., Youn D.-Y., Lee H., Lee C.H. (2014). A high-throughput assay of NK cell activity in whole blood and its clinical application. Biochem. Biophys. Res. Commun..

[9] Jobin G., Rodriguez-Suarez R., Betito K. (2017). Association between natural killer cell activity and colorectal cancer in high-risk subjects undergoing colonoscopy. Gastroenterology.

[10] Barkin J., Rodriguez-Suarez R., Betito K. (2017). Association between natural killer cell activity and prostate cancer: A pilot study. Can. J. Urol..

[11] Choi S.I., Lee S.H., Park J.-Y., Kim K.-A., Lee E.J., Lee S.Y., In K.H. (2019). Clinical utility of a novel natural killer cell activity assay for diagnosing non-small cell lung cancer: A prospective pilot study. Onco Targets Ther..

[12] Oh S., Chun S., Hwang S., Kim J., Cho Y., Lee J., Kwack K., Choi S.-W. (2021). Vitamin D and Exercise Are Major Determinants of Natural Killer Cell Activity, Which Is Age- and Gender-Specific. Front. Immunol..

[13] Harrison M. (2015). Erythrocyte sedimentation rate and C-reactive protein. Aust. Prescr..

[14] Pepys M.B., Hirschfield G.M. (2003). C-reactive protein: A critical update. J. Clin. Investig..

[15] Spiegel A., Brooks M.W., Houshyar S., Reinhardt F., Ardolino M., Fessler E., Chen M.B., Krall J.A., DeCock J., Zervantonakis I.K. (2016). Neutrophils suppress intraluminal NK cell–mediated tumor cell clearance and enhance extravasation of disseminated carcinoma cells. Cancer Discov..

[16] Jaeger B.N., Donadieu J., Cognet C., Bernat C., Ordoñez-Rueda D., Barlogis V., Mahlaoui N., Fenis A., Narni-Mancinelli E., Beaupain B. (2012). Neutrophil depletion impairs natural killer cell maturation, function, and homeostasis. J. Exp. Med..

[17] Barker T., Fulde G., Moulton B., Nadauld L.D., Rhodes T. (2020). An elevated neutrophil-to-lymphocyte ratio associates with weight loss and cachexia in cancer. Sci. Rep..

[18] Howard R., Kanetsky P.A., Egan K.M. (2019). Exploring the prognostic value of the neutrophil-to-lymphocyte ratio in cancer. Sci. Rep..

[19] Alkhatip A., Kamel M.G., Hamza M.K., Farag E.M., Yassin H.M., Elayashy M., Naguib A.A., Wagih M., Abd-Elhay F.A.-E., Algameel H.Z. (2021). The diagnostic and prognostic role of neutrophil-to-lymphocyte ratio in COVID-19: A systematic review and meta-analysis. Expert Rev. Mol. Diagn..

[20] Wang X., Zhang G., Jiang X., Zhu H., Lu Z., Xu L. (2014). Neutrophil to lymphocyte ratio in relation to risk of all-cause mortality and cardiovascular events among patients undergoing angiography or cardiac revascularization: A meta-analysis of observational studies. Atherosclerosis.

[21] Kim B.-R., Chun S., Cho D., Kim K.-H. (2019). Association of neutrophil-to-lymphocyte ratio and natural killer cell activity revealed by measurement of interferon-gamma levels in a healthy population. J. Clin. Lab. Anal..

[22] Nederby L., Jakobsen A., Hokland M., Hansen T.F. (2018). Quantification of NK cell activity using whole blood: Methodological aspects of a new test. J. Immunol. Methods.

[23] Lee J., Park K.H., Ryu J.H., Bae H.J., Choi A., Lee H., Lim J., Han K., Park C.H., Jung E.S. (2017). Natural killer cell activity for IFN-gamma production as a supportive diagnostic marker for gastric cancer. Oncotarget.

[24] Vano Y.-A., Oudard S., By M.-A., Têtu P., Thibault C., Aboudagga H., Scotté F., ElAidi R. (2018). Optimal cut-off for neutrophil-to-lymphocyte ratio: Fact or fantasy? A prospective cohort study in metastatic cancer patients. PLoS ONE.

[25] Templeton A.J., McNamara M.G., Šeruga B., Vera-Badillo F.E., Aneja P., Ocaña A., Leibowitz-Amit R., Sonpavde G., Knox J.J., Tran B. (2014). Prognostic role of neutrophil-to-lymphocyte ratio in solid tumors: A systematic review and meta-analysis. J. Natl. Cancer Inst..

[26] Campos C., Pera A., Lopez-Fernandez I., Alonso C., Tarazona R., Solana R. (2014). Proinflammatory status influences NK cells subsets in the elderly. Immunol. Lett..

[27] Mocikat R., Braumüller H., Gumy A., Egeter O., Ziegler H., Reusch U., Bubeck A., Louis J., Mailhammer R., Riethmüller G. (2003). Natural killer cells activated by MHC class I low targets prime dendritic cells to induce protective CD8 T cell responses. Immunity.

[28] Gaudino S.J., Kumar P. (2019). Cross-Talk between Antigen Presenting Cells and T Cells Impacts Intestinal Homeostasis, Bacterial Infections, and Tumorigenesis. Front. Immunol..

[29] Maher S., Romero-Weaver A., Scarzello A., Gamero A. (2007). Interferon: Cellular executioner or white knight?. Curr. Med. Chem..

[30] Paul S., Lal G. (2017). The Molecular Mechanism of Natural Killer Cells Function and Its Importance in Cancer Immunotherapy. Front. Immunol..

[31] Guthrie G.J.K., Charles K.A., Roxburgh C.S.D., Horgan P.G., McMillan D.C., Clarke S.J. (2013). The systemic inflammation-based neutrophil–lymphocyte ratio: Experience in patients with cancer. Crit. Rev. Oncol. Hematol..

[32] Imai K., Matsuyama S., Miyake S., Suga K., Nakachi K. (2000). Natural cytotoxic activity of peripheral-blood lymphocytes and cancer incidence: An 11-year follow-up study of a general population. Lancet.

[33] Liu J., Liu Y., Xiang P., Pu L., Xiong H., Li C., Zhang M., Tan J., Xu Y., Song R. (2020). Neutrophil-to-lymphocyte ratio predicts critical illness patients with 2019 coronavirus disease in the early stage. J. Transl. Med..

[34] Huang C., Wang Y., Li X., Ren L., Zhao J., Hu Y., Zhang L., Fan G., Xu J., Gu X. (2020). Clinical features of patients infected with 2019 novel coronavirus in Wuhan, China. Lancet.

[35] Osman M., Faridi R.M., Sligl W., Shabani-Rad M.-T., Dharmani-Khan P., Parker A., Kalra A., Tripathi M.B., Storek J., Tervaert J.W.C. (2020). Impaired natural killer cell counts and cytolytic activity in patients with severe COVID-19. Blood Adv..

[36] Van Eeden C., Khan L., Osman M.S., Tervaert J.W.C. (2020). Natural Killer Cell Dysfunction and Its Role in COVID-19. Int. J. Mol. Sci..

[37] Jonasson L., Backteman K., Ernerudh J. (2005). Loss of natural killer cell activity in patients with coronary artery disease. Atherosclerosis.

[38] Ong S., Rose N.R., Čiháková D. (2017). Natural killer cells in inflammatory heart disease. Clin. Immunol..

[39] Feldman M., Aziz B., Kang G.N., Opondo M.A., Belz R.K., Sellers C. (2013). C-reactive protein and erythrocyte sedimentation rate discordance: Frequency and causes in adults. Transl. Res..

[40] Oda E., Kawai R. (2010). Comparison between high-sensitivity C-reactive protein (hs-CRP) and white blood cell count (WBC) as an inflammatory component of metabolic syndrome in Japanese. Intern. Med..

[41] Willems J.M., Trompet S., Blauw G.J., Westendorp R.G.J., de Craen A.J.M. (2010). White blood cell count and C-reactive protein are independent predictors of mortality in the oldest old. J. Gerontol. A Biol. Sci. Med. Sci..

